# Social support and the self-rated health of older people: A comparative study in Tainan Taiwan and Fuzhou Fujian province

**DOI:** 10.1097/MD.0000000000003881

**Published:** 2016-06-17

**Authors:** Yue Dai, Chen-Yun Zhang, Bao-Quan Zhang, Zhanzhan Li, Caixiao Jiang, Hui-Ling Huang

**Affiliations:** a Department of Health Management, School of Public Health, Fujian Medical University, Fuzhou Fujian Province; b Department of Healthcare Management, School of Public Health, Central South University, Changsha Hunan Province; c Department of Health Policy and Law, School of Public Health, Fujian Medical University; d Department of Neonatology, Fujian Maternal and Child Health, Fuzhou Fujian Province; e Department of Epidemiology and Statistics; f Department of Epidemiology and Statistics, School of Public Health, Central South University, Changsha Hunan Province; g Department of Gerontology, Union Hospital of Fujian Medical University, Fuzhou Fujian Province, China.

**Keywords:** Fuzhou, older people, social support, SRH, Tainan

## Abstract

The lack of social support in elderly populations incurs real societal costs and can lead to their poor health. The aim of this study is to investigate the self-rated health (SRH) and social support among older people as well as its associated factors.

We conducted a cross-sectional study among 312 urban community-dwelling elderly aged 65 to 90 years in Tainan Taiwan and Fuzhou Fujian Province from March 2012 to October 2012. A Spearson correlation test, independent *t* test, a Pearson *χ*^2^ test, a linear regression analysis, and a multiple-level model were performed to analyze the results.

The participants identified children as the most important source of objective and subjective support, followed by spouse and relatives. Tainan's elderly received more daily life assistance and emotional support, showed stronger awareness of the need to seek help, and maintained a higher frequency of social interactions compared with the elderly in Fuzhou. The mean objective support, subjective support, and support utilization scores as well as the overall social support among Tainan's elderly were significantly high compared with the scores among Fuzhou's elderly. Further, Tainan's elderly rated better SRH than Fuzhou's elderly. Correlation analysis showed that social support was significantly correlated with city, age, living conditions, marital status, and SRH. Multiple linear regression analysis, with social support as a dependent variable, retained the following independent predictors in the final regression model: city (4.792, 95% confidence interval [CI]: 3.068–6.516, *P* = 0.000), age (−0.805, 95% CI: −1.394 to −0.135, *P* = 0.013), marital status (−1.260, 95% CI: −1.891 to −0.629, *P* = 0.000), living conditions (4.069, 95% CI: 3.022–5.116, *P* = 0.000), and SRH −1.941, 95% CI: −3.194 to −0.688, *P* = 0.003). The multiple-level model showed that city would impact older people's social support (*χ*^2^ = 5.103, *P* < 0.001). Marital status (−2.133, 95% CI: −2.768 to −1.499, *P* = 0.000), education (1.697, 95% CI: 0.589–2.805 *P* = 0.003), living conditions (4.20, 95% CI: 1.762–6.638, *P* = 0.000), and SRH (−3.144, 95% CI: −4.502 to −1.727, *P* = 0.000) were the associated factors. Thus, city, age, marital status, education, living conditions, and SRH might be the associated factors for social support among older people.

This study presents some feasible implications for social support improvement in China and in other nations worldwide.

## 1 Introduction

Aging is associated with an increased reliance on health-related and support services.^[1]^ Old age often goes hand in hand with increasingly complex and often interrelated problems, encompassing physical, psychological, and social health.^[2,3]^ In 2007, 11% of the population worldwide was 60 years of age or older; this figure is estimated to increase to 22% by 2050.^[4]^ Most public policy debates are concerned with the physical issues of aging, whereas social issues, such as social support, tend to be ignored.^[5]^ Older people are faced with greater losses, given fewer social resources and less adequate social support, in both subjectively perceived support and the frequency of contact.^[6]^ Physical activity (PA) also plays a key role in maintaining health and mobility in old age^[7–9]^; the evidence for the health benefits of PA is stronger for adults 65 years and older than for any other age group because the consequences of inactivity are more severe for this age group. Furthermore, older people with a high-level social support may achieve the recommended PA more easily than those with lower social support levels, thereby maintaining health and physical function.^[10]^

Social support consists of addressing tangible needs, such as assistance with transportation, home and personal care, as well as emotional support such as being listened to, understood, and comforted.^[11]^ Social support has been recognized as an important social determinant of health because it assists individuals in reaching their physical and emotional needs, and it reduces the effects of stressful events on their quality of life.^[12]^ More recently, many studies have demonstrated a relation between social support and health including mortality, chronic diseases, cognition, depressive symptoms, and well-being.^[13–15]^ Self-rated health (SRH) is often considered to be a valid, reliable, and robust measure of health as well as a predictor of mortality among older people.^[16]^ Associations between low social support and poor perceived health, including health-related quality of life and SRH, have also been demonstrated.^[17–19]^ Therefore, interventions that target social support may be a priority to improve the well-being of older people and maximize their health and functional capacity.

Considering the significant role of social support in later life, the present study investigates social support and SRH, as well as the factors associated with social support in contemporary Chinese older people. Specifically, we report our survey findings regarding the elderly Chinese population in Tainan and Fuzhou. Both cities are populated by Chinese residents; however, they differ in their socioeconomic development and in the social organization of work and community life. We believe that a comparison of Tainan and Fuzhou is important because it will help us to assess the impact of macrosocial structures on the social support and health of elderly people. Thus, our specific concerns are to investigate social support and SRH; to identify the associated factors that most contribute to the social support of the elderly; and to analyze the similarities and differences in the social support available to the elderly between the 2 cities.

## 2 Methods

### 2.1 Ethical approval

This study was approved by the institutional review boards of Fujian Medical University. All participants in the present study gave their informed consent before their inclusion in the study. Details that might disclose the identity of the subjects under study were omitted and all data collected were unidentified.

### 2.2 Setting

Both Tainan and Fuzhou are Chinese cities that belong to The People's Republic of China, but represent “two systems”.^[20]^ Taiwan was formerly under the colonial rule of the Netherlands; modern-day Tainan is a city located in southwestern Taiwan at 120.2 degree East and 23.0 degree North. Taiwan covers a total area of 846.20 square miles (2191.65 km^2^) and is bordered by the Taiwan Strait to the west. In 2011, the total population of Tainan was 1,882,000, and elderly people aged 65 years or older comprised 11.69% of the population.^[21]^ Fisheries, agriculture, manufacturing, and high technology are the city's main industries. In addition, the local per capita gross domestic product (PCGDP) was approximately USD 18,390.01, and the per capita disposable income (PCDI) was USD 8268.24 in 2012.^[21]^ Over the last half century, Taiwan has emerged as a highly modernized commercial center in East Asia.

Fuzhou (119.3 degree East, 26.08 degree North), with an area of 4620.87 square miles (11.968 km^2^) and a population of 7,115,370, is the political and economic center of Fujian Province. Fuzhou is located on the northwestern coast of the Taiwan Strait and consists of 5 urban districts (the Gulou, Taijiang, Jin’an, Cangshan, and Mawei Districts) and 8 counties. In 2010, 860,104 people in Fuzhou were 60 years of age and older, accounting for 12.09% of the total population, and 584,126 people were 65 years of age and older, accounting for 8.21% of the total population.^[22]^ The city's people are diverse in many respects, and there are 43 ethnic groups. At the end of 2012, the local PCGDP was approximately USD 9231.29, and the PCDI was USD 4522.92.^[22]^ It should be noted that most of the local people living in contemporary Taiwanese cities are the offsprings of the people who formerly lived in mainland China, particularly of the people from Fujian Province.^[23]^

### 2.3 Participants

**Figure 1:**
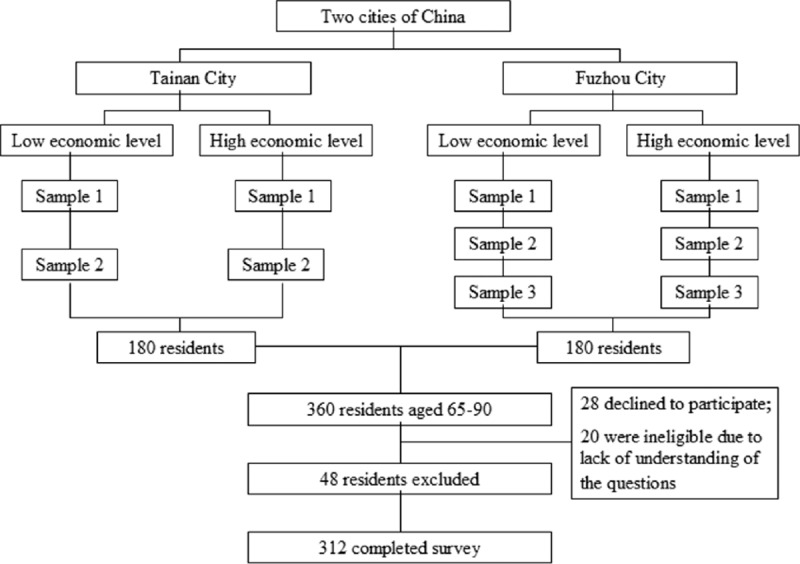
A multistage stratified random cluster sampling technique was used to recruit the sample. In Tainan and Fuzhou, 10 administrative districts were randomly selected from these districts to achieve the desired sample size. A total of 312 older people completed survey.

This was a cross-sectional study. The target population was residents aged 65 years and older, living in local urban communities for over 6 months, and was able to communicate in the Mandarin or Minnan dialects. The survey was conducted from March 2012 to October 2012. We used multistage stratified random cluster sampling method to collect the sample. The sample size was calculated based on the proportion of older people within the total population in the 2 cities in 2011. According to the layout of Tainan and Fuzhou, we randomly selected several communities from each administrative district. In Tainan, 4 administrative districts were stratified into low- and high-economic-level groups, and 1 or 2 districts was randomly selected from each group, a total of 6 communities (low economic level: n = 3; high economic level: n = 3) were selected from these districts. In Fuzhou, 6 administrative districts were stratified into low- and high-economic-level groups, and 2 districts were randomly selected from each group, a total of 8 communities (low economic level: n = 4; high economic level: n = 4) were selected. Finally, 360 older people were selected from Tainan and Fuzhou (Fig. 1). Data were collected by self-designed questionnaire including sociodemographic information and Social Support Rate Scale (SSRS).

### 2.4 Instruments

SRH was assessed using 1 question: “Compared with other people of your age, how do you rate your health?” Responses were given with a 3-point scale of good, fair, poor. We also developed a health checklist that evaluated the prevalence of chronic diseases, 2-week outpatient clinic visits, and the average duration of hospitalization to better understand the health status of the participants.

The SSRS was used to measure social support.^[24]^ This scale is composed of 3 dimensions, namely, objective support, subjective support, and support utilization. Objective support reflects individual social networks and actual received instrumental and emotional support in the past, which is measured by 3 items: (2) your living conditions in a recent year, (6) your source of economic support during an emergency situation, and (7) your source of comfort and care during an emergency situation. Subjective support represents individual subjective perceptions, such as the extent of being respected and understood, which is measured by 4 items: (1) the number of close friends that are able to help you, (3) your relationship with your neighbors, (4) your relationship with your colleagues, and (5) the degree of support or care from family members. Support utilization explains how to seek and use his/her social support, which is measured by 3 items: (8) the way you pour out your feelings when you encounter difficulties, (9) the way you seek help when you encounter difficulties, and (10) the frequency with which you participate in social activities.^[25]^ The SSRS consists of 10 items, with scores ranging from 1 to 4, and the total score ranges from 12 to 65. Higher score indicates a higher level of social support. The Cronbach's α coefficient of this scale was 0.89–0.94 and a test-retest reliability was 0.92. The predictive validity was high.^[26]^

The participants’ sociodemographic information was also collected, including age, sex, marital status, living conditions, previous employment, education, welfare, among others.

### 2.5 Statistical analysis

The data were summarized as frequencies or percentages (%) for categorical variables (age, sex, ethnicity, education, health insurance, living conditions, marital status, previous employment, and SRH) and as means, standard deviations, or ranges for continuous variables (overall social support, subjective support, objective support, and support utilization scores) for analysis. The normality of continuous variables was checked by the Kolmogorov–Smirnov test. The independent *t* test and Mann–Whitney *U* test were used to compare continuous variables when the distribution was normal, and the analysis of variance (ANOVA) and Kruskal–Wallis test were used when the distribution was non-normal. Categorical data were analyzed by the Pearson *χ*^2^ test when appropriate.

We conducted further analyses to investigate the association between social support and sociodemographic variables. First, the correlation between social support and these variables was assessed using Spearson correlation coefficients. Univariate analysis was also used to explore the possible associated factors. Second, we performed multiple linear regression analyses with social support as the dependent variable and all sociodemographic variables as independent variables. Collinearity diagnostics were also conducted using multiple linear regressions. Bidirectional stepwise regression analysis was performed using a *P* value-to-remove of 0.1 and a *P* value-to-enter of 0.05 to assess which variable was most useful in the estimation of social support. A full regression model was also established. Finally, based on previous research findings and theoretical considerations, we ran a multiple-level model not only to determine the effects of individual- (sociodemographic) and city-level variables on older people's social support but also to retest the results of the multiple linear regression. The multiple-level modeling followed a stepwise process. The first step added the city variable to the null model to determine whether the city impacts older people’ social support. The second step included all individual variables to determine which variables were significantly associated with social support. Accordingly, all individual variables were treated as fixed effects and random effects. Linear regression analyses were performed using SPSS v.21.0 (IBM, Armonk, NY). Multiple-level analyses were conducted in MLwiN 6.40 (Centre for Multilevel Modeling, University of Bristol, Bristol, UK).

## 3 Results

### 3.1 Participant characteristics and health status

Among the 360 potential participants, 28 declined to participate, 20 were ineligible because of an inability to understand the questions, and 312 completed the questionnaire, which yielded a response rate of 86.67%. The median age of the participants was 72.1 (range 65–90), with more female (51.92%) than male (48.08%) participants. Most participants were of Han ethnicity (98.5%), married (68.91%), and nonmanual workers (57.37%), with low education (middle school and below: 72.75%); 96.79% of the sample had ≥1 types of healthcare insurance. Most participants rated their health as fair (62.82%). After sociodemographic standardization, 22.11% of Tainan's elderly suffered from at least 1 type of chronic disease compared with 33.33% in Fuzhou. The 2 most prevalent chronic diseases were high blood pressure and diabetes mellitus. The 2-week prevalence of illness in Tainan was 16.44% and the average number of clinic visits related to the disease was 0.36 times per person; these rates were 21.25% and 0.31, respectively, in Fuzhou. The participant's demographic characteristics and health status are summarized in Table 1.

**Table 1:**
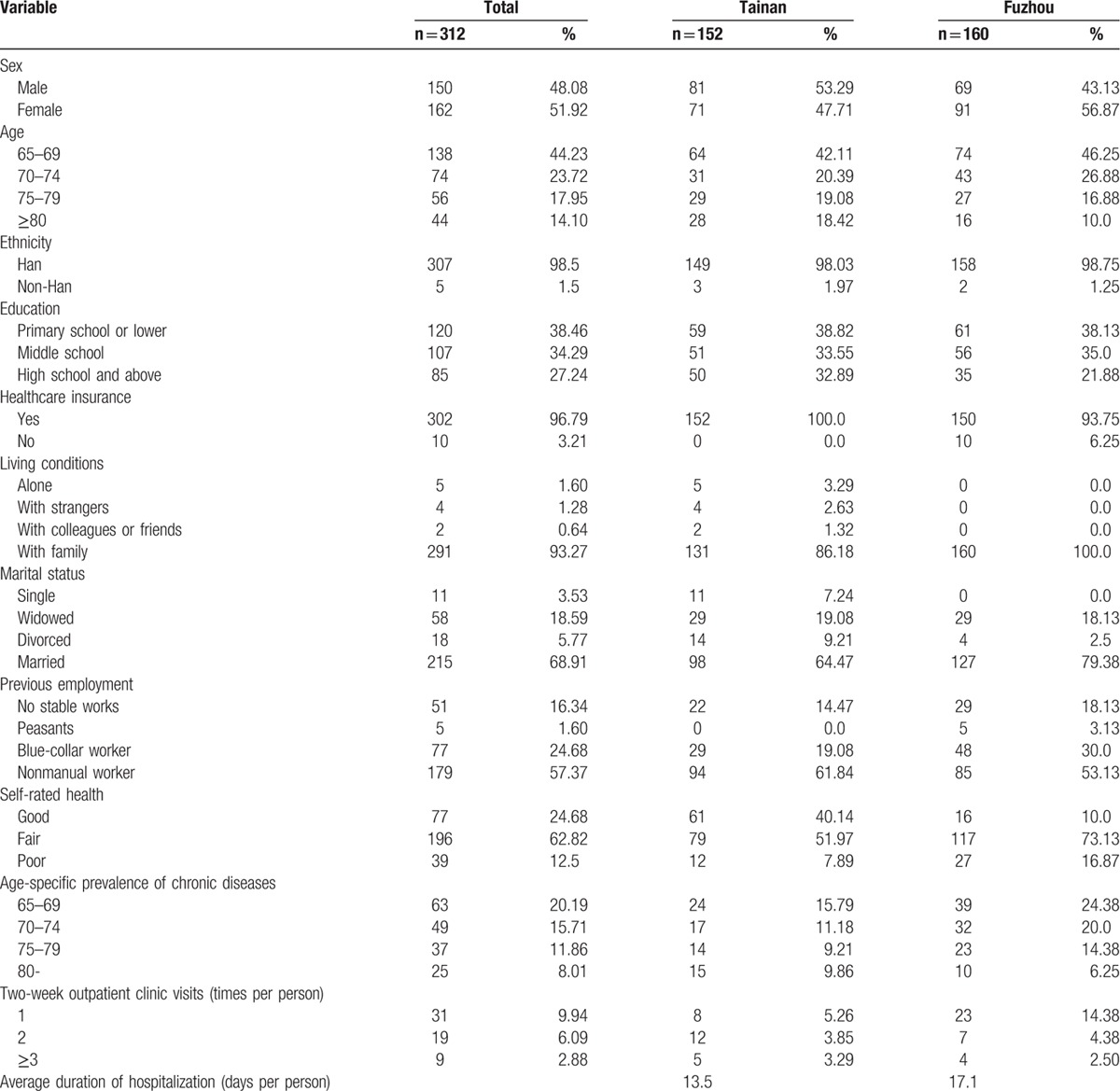
Sociodemographic characteristics and health status of the participants.

### 3.2 The social support of the participant

The mean overall social support was 36.54 ± 7.38 out of a range of 17 to 54. The mean scores for objective support, subjective support, and support utilization were 9.38 ± 2.83, 20.52 ± 4.06, and 6.65 ± 2.04, respectively, out of the ranges of 1 to 18, 11 to 30, and 3 to 11. Among Tainan's older people, the mean overall social support was 38.17 ± 8.36 out of a range of 17 to 54. The mean objective support, subjective support, and support utilization scores were 9.66 ± 3.57, 21.23 ± 4.22, and 7.17 ± 2.09, respectively, out of the ranges of 3 to 15, 11 to 30, and 4 to 12. Among Fuzhou's older people, the mean overall social support was 34.99 ± 5.94 out of a range of 14 to 52. The mean objective support, subjective support, and support utilization were 9.10 ± 1.89, 19.74 ± 3.57, and 6.15 ± 1.87, respectively, out of the ranges of 2 to 14, 11 to 29, and 3 to 11 (Table 2). Compared with the Chinese norm (34.56 ± 3.37, n = 128),^[27]^ the overall social support of Tainan's older people was significantly higher (*t* = 5.323, *P* < 0.001). The participants with good SRH reported high social support level (Table 2).

**Table 2:**
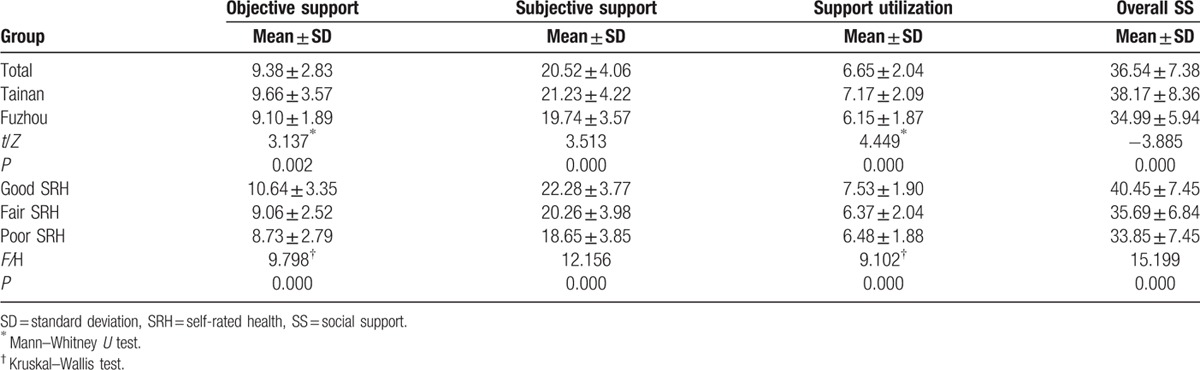
Social support scores of the participants.

In both cities, children were identified as the most important source of instrumental support and emotional support, followed by spouse and relatives (Tables 3 and 4). More than half of the participants (n = 205, 65.70%) reported having 1 or 2 close friends who could support them. Most participants perceived their children (89.1%, n = 278) and spouse (70.5%, n = 220) as the primary source of subjective support. Nearly 40% (n = 123) of the participants sometimes or always asked for help, and 48.07% (n = 150) sometimes or always confided in someone when they were in trouble. Nearly half (n = 75, 49.34%) of Tainan's elderly often or always attended social activities, but more than two-thirds (n = 115, 71.88%) of the Fuzhou participants seldom or never participated in social activities (Table 5).

**Table 3:**
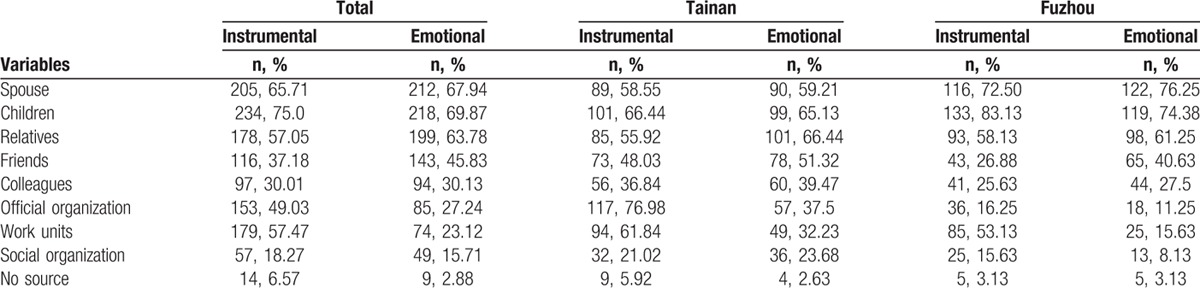
The objective support of the participants.

**Table 4:**
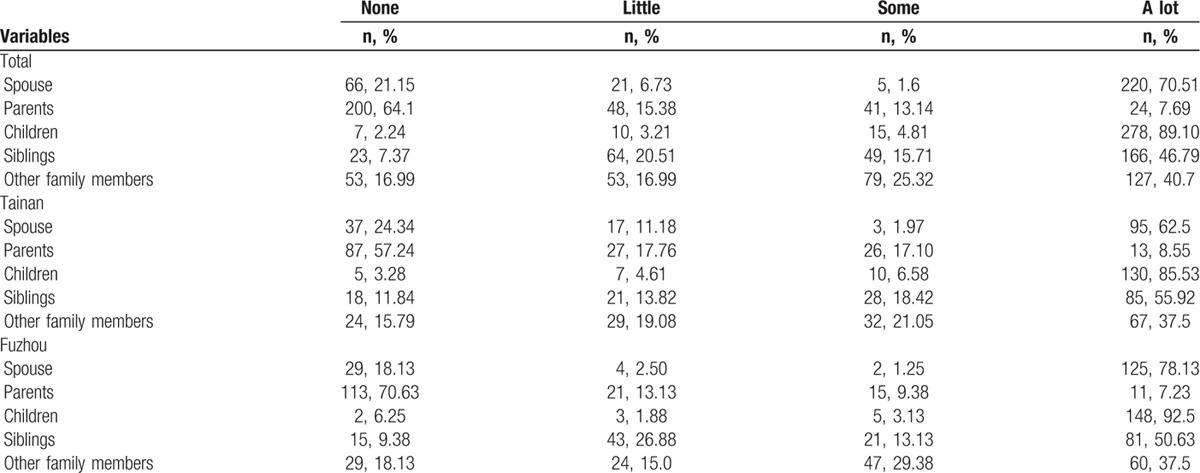
The subjective support of the participants.

**Table 5:**
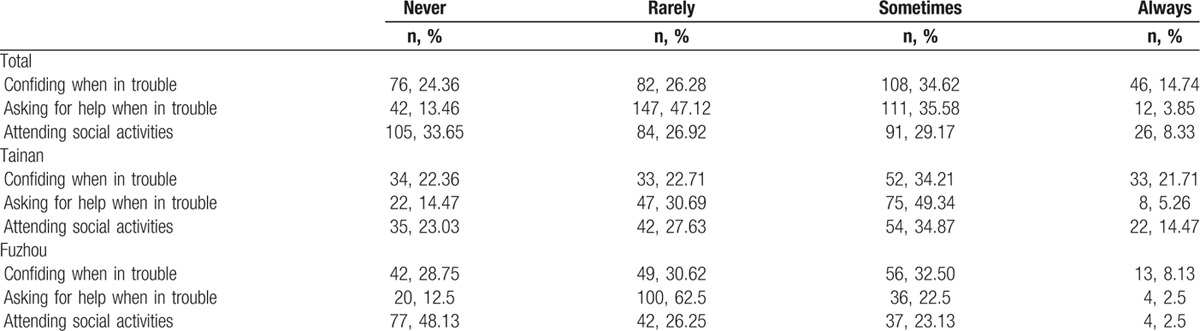
The support utilization of the participants.

### 3.3 Factors associated with social support

**Figure 2:**
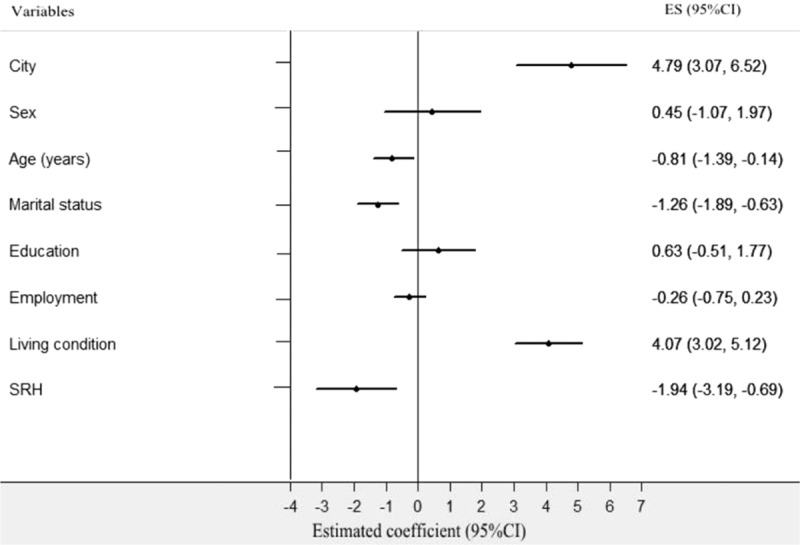
Multiple linear regression analysis of associated factors for social support in older people. Social support as a dependent variable, the following independent predictors retained in the final regression model: city, age, marital status, living condition, and SRH.

**Figure 3:**
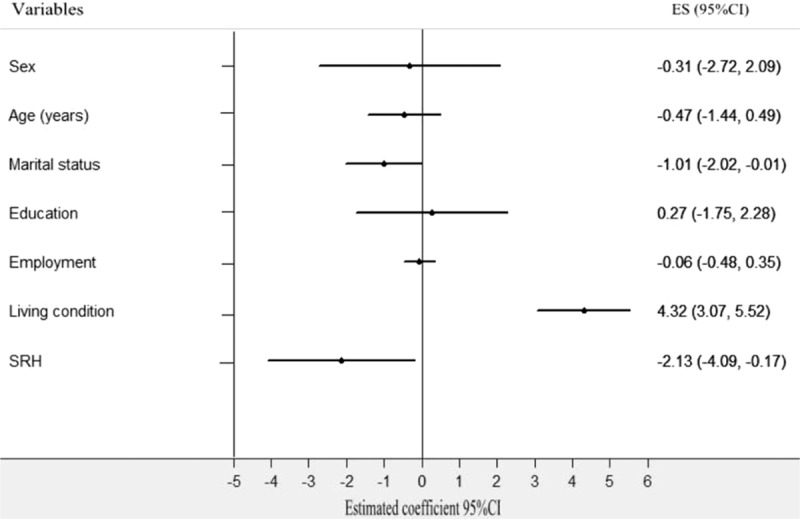
Multiple linear regression analysis of associated factors for social support in Tainan's older people. Social support as a dependent variable, the following independent predictors retained in the final regression model: marital status, living condition, and SRH.

**Figure 4:**
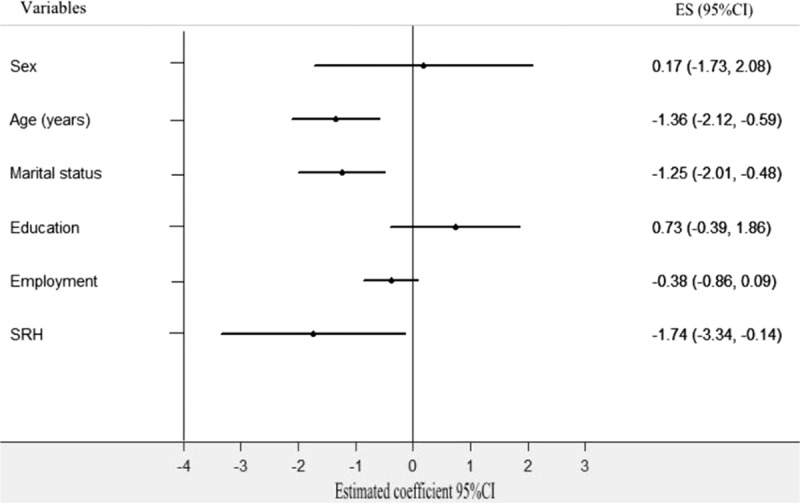
Multiple linear regression analysis of associated factors for social support in Fuzhou's older people. Social support as a dependent variable, the following independent predictors retained in the final regression model: age, marital status, and SRH.

First, the Spearson correlation analysis revealed that social support was significantly correlated with city (*r* = 0.215, *P* < 0.001), age (*r* = −0.233, *P* < 0.001), living conditions (*r* = 0.362, *P* < 0.001), marital status (r = −0.326, *P* < 0.001), and SRH (*r* = −0.285, *P* < 0.001). Meanwhile, the univariate analysis indicated that overall social support was significantly different by city group, age group, marital status group, living condition group, and SRH group (Table 6). Second, a stepwise multiple linear regression analysis was conducted. City (4.792, 95% confidence interval [CI]: 3.068–6.516, *P* = 0.000), age (−0.805, 95% CI: −1.394 to −0.135, *P* = 0.013), marital status (−1.260, 95% CI: −1.891 to −0.629, *P* = 0.000), living conditions (4.069, 95% CI: 3.022–5.116, *P* = 0.000), and SRH (−1.941, CI: −3.194 to −0.688, *P* = 0.003) were each independent correlates of overall social support. The variance inflation factor (VIF) of all sociodemographic variables was <2.0, which indicated that there was not significant collinearity among sociodemographic variables. Thus, the equation for social support was built using multiple linear regression: social support = 20.07 + 4.792 City-0.805 Age-1.260 Marital status + 4.096 Living conditions-1.955 SRH. *R* = 0.697 (Table 7 and Fig. 2). Third, according to the city classification, we conducted linear regression analyses of social support to find the differences in associated factors for the 2 cities. The results showed that marital status (−1.012, 95% CI: −2.017 to −0.008, *P* = 0.000), living conditions (4.324, 95% CI: 3.067–5.518, *P* = 0.000), and SRH (−2.128, 95% CI: −4.087 to −0.170, *P* = 0.000) were the associated factors of social support among Tainan's older people (Table 8 and Fig. 3). Age (−1.335, 95% CI: −2.121 to −0.588, *P* = 0.001), marital status (−1.246, CI: −2.008 to −0.484, *P* = 0.001), and SRH (−1.74, CI: −3.337 to −0.144, *P* = 0.033) influenced the social support of Fuzhou's older people (Table 8 and Fig. 4).

**Table 6:**
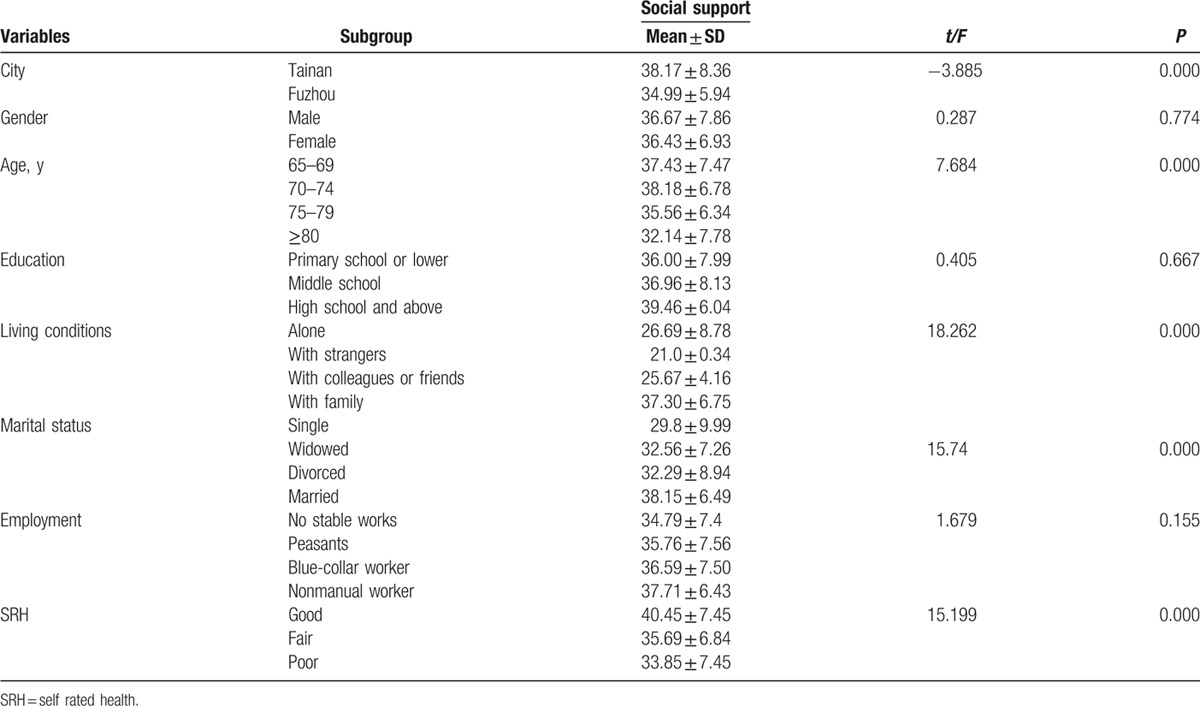
Univariate analyses of associated factors for social support in older people.

**Table 7:**
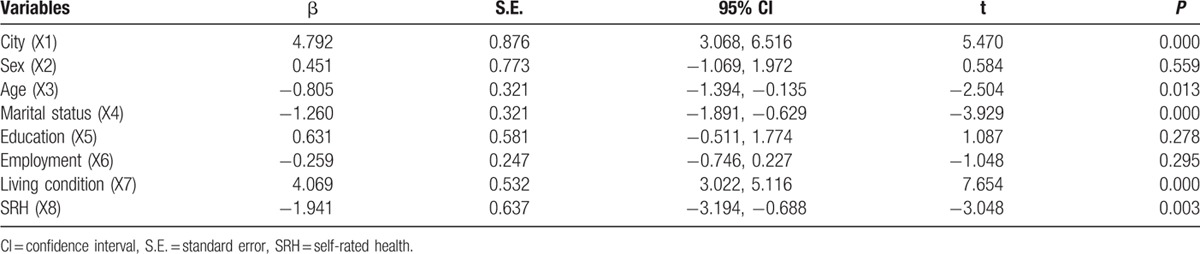
Multiple linear regression analysis of associated factors for social support in older people.

**Table 8:**
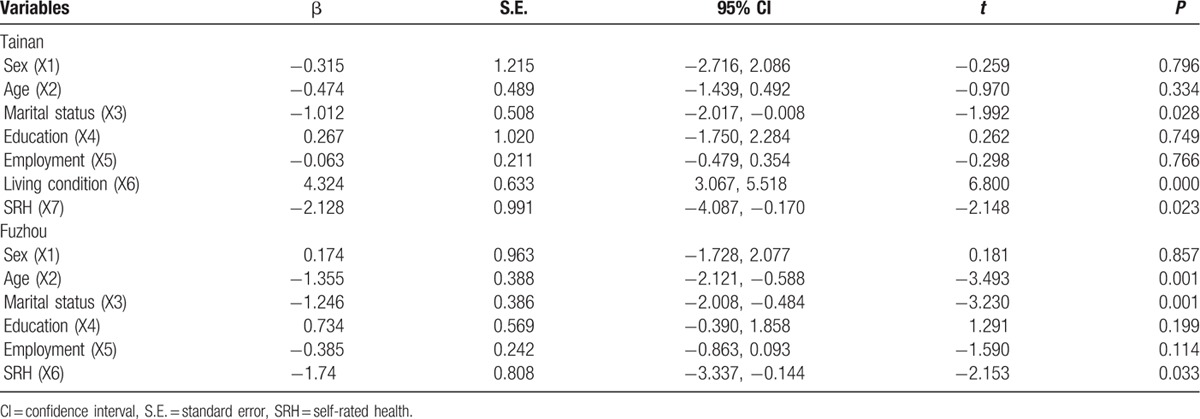
Respective multiple linear regression analysis of associated factors for social support in older people.

**Figure 5:**
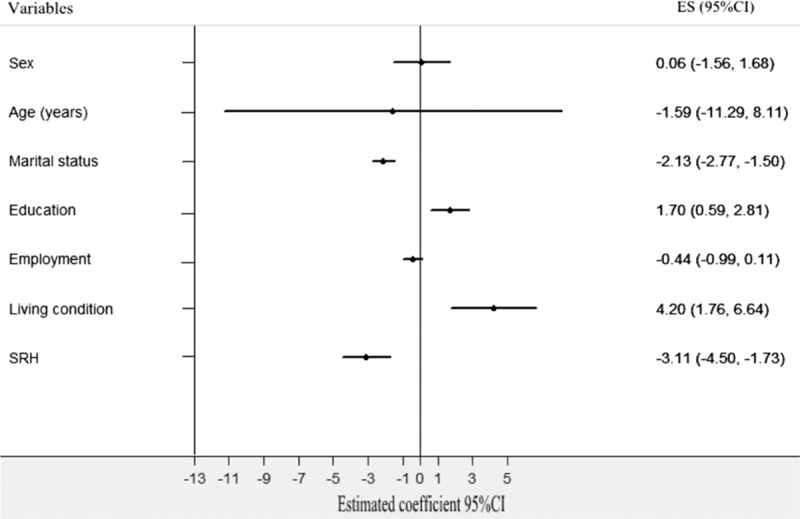
Multiple-level model analysis of associated factors for social support in older people. Marital status, education, living condition, and SRH were the associated factors of social support in older people.

Finally, because city had the most significant impact on the social support of older people, we ran a multiple-level model to adjust the city-level variable. The null model without adjustment for covariates indicated that social support was significantly different (*χ*^2^ = 5.103, *P* < 0.001) when differences between cities were taken into consideration without other sociodemographic variables. Marital status (−2.133, CI: −2.768 to −1.499, *P* = 0.000), education (1.697, 95% CI: 0.589–2.805 *P* = 0.003), living conditions (4.20, 95% CI: 1.762–6.638, *P* = 0.000), and SRH (−3.144, 95% CI: −4.502 to −1.727, *P* = 0.000) were associated with social support in the full multiple-level model (Table 9 and Fig. 5). The final results from the full multiple-level model including the city-level difference were nearly consistent with the results of the linear regression model, with a β similar or slightly larger than the corresponding β in the linear regression analyses. Thus, city, age, marital status, education, living conditions, and SRH were found to be the associated factors of social support among older people.

**Table 9:**
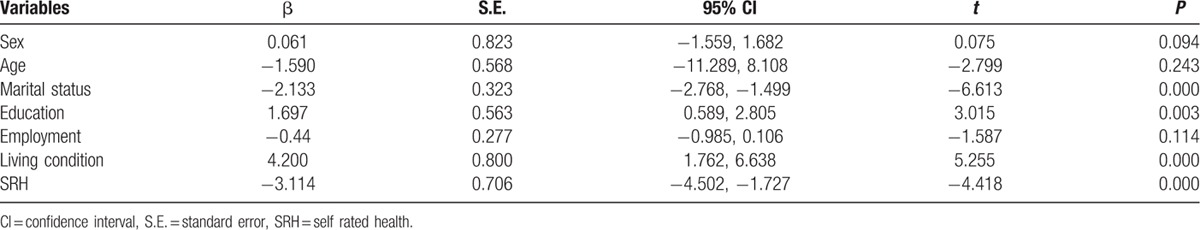
Multiple-level model analysis of associated factors for social support in older people.

## 4 Discussion

### 4.1 The social support of older people

The social support level of Tainan's older people is higher than that of Fuzhou's elderly, which led us to identify what caused this difference between the 2 cities. First, the social network structure may influence social support through its mediating function. For instance, support from friends or neighbors is particularly important for older people because it is flexible and provides a better opportunity to be understood and share experiences.^[28]^ Personal social networks may provide social companionship, instrumental aid, and emotional comfort to the elderly, which helps to alleviate pressure, reduce depressive feelings, and moderate the ill effects of stressful life events on health.^[29]^

Second, community participation and engagement in social activities help elderly people to remain connected to society.^[30]^ Lin et al^[31]^ found that 1 important factor that influenced the successful aging of Taiwan's elderly population was participation in social activities. Chen et al^[32]^ reported that social activitiesin which the elderly were engaged had increased in Taiwan in recent years, as had their social support and satisfaction with life.

Third, support utilization should be considered because people may not use their support effectively even being provided with important support resources. As asserted by Taylor et al,^[33]^ maintaining harmony in social groups was one of the significant norms in Asian culture, many people thought that seeking help might affect the friendly relationship between each other. But it was opposite in Western culture. Because of the different social systems, Tainan's older people may be influenced more by Western culture than Fuzhou's elderly, which could increase their willingness to seek help.

### 4.2 The relationship between social support and SRH

As suggested in previous research, the perception of having adequate social support is associated with better SRH among community-dwelling older persons.^[34]^ Our findings are also supported by several empirical studies highlighting the association between social connectedness and SRH.^[35,36]^ For instance, a comparative study conducted among older people in Western Finland and Northern Sweden showed that individual-level social capital including social participation was significantly associated with SRH.^[37]^ Another cross-sectional study involving 2731 participants in Japan concluded that participants with higher SRH had sufficient social support.^[38]^ Because social support is regarded as the mechanism that links social capital and health outcomes,^[39]^ the association between social support and SRH may be either direct (e.g., provision of health information) or indirect (e.g., help with a job search, which promotes health).^[40]^ Furthermore, individual features of social support can be considered to be a resource for the health and well-being of older people.^[41]^

### 4.3 The associated factors of social support and health

Available literatures, despite differences in research designs and targeted populations, suggest that to understand the social support and health of older people, special attention should be paid to their socioeconomic status (SES),^[42–44]^ which is measured by income, wealth, education, work, social insurance, SRH, out-of-pocket costs, and transportation to health facilities.^[45,46]^ Several studies have to date found the health of older people in China is also influenced by demographic characteristics and SES, such as age, sex, marital status, living condition, chronic diseases.^[47,48]^

Results of this study showed that social support is associated with age, education, marital status, and living conditions. First, older people who have a higher educational level may have better communication ability and interpersonal skills so that they can utilize support resources actively. In addition, although social security system has been well established in rural areas in mainland China, most older people are not familiar with the social insurance because of their poor literacy, which may lead to the benefits of social security they received very low.^[49]^

Second, the widowed, single, and divorced elderly experience poor social support compared with the married elderly; possible explanations may be the loss of the source of support or the negative effects of living alone. With a healthier marital status, older people may tend to perceive that more social support is available to them, and they may be able to build and maintain larger social networks.^[50]^ Hewitt et al^[51]^ revealed the moderating effect of marital status on social support, especially for men who became widowed, among older people. However, Penning et al^[52]^ found that marital status is not uniformly positive, neutral, or negative in terms of its implications for extra-household social support among older people. Thus, a longitudinal design is needed to investigate the influence of marital status on social support of the elderly.

Third, because the influence of Confucian cultural traditions, social life in 2 cities is predominantly family-oriented, close kin relationships and familial interests predominate, those living alone appear to be or perceive themselves as the most vulnerable group compared with the other living conditions.^[53–55]^ For instance, even if they confront the same medical conditions as other groups, the absence of caregiver in their daily life may make it more difficult for older people to cope with the incapacitating effects of illness. Additionally, the one-child policy has been in place for nearly 40 years in mainland China, which would cause great difficulties in living conditions for older people.

Finally, age is an associated factor of social support among Fuzhou's elderly, which suggests a comparison of the differences in the macrosocial environments.^[56,57]^ In contrast to mainland China, Taiwan has launched a series of senior service programs and senior health promotion measures, for instance, the “Aging Welfare Program” in 1999, the new labor pension system and community care centers in 2005, the Mega Warmth Social Welfare Plan in 2006, and Age-Friendly Cities in 2008.^[58]^ Taiwan has already established ideal aging policies that greatly support the participation of elderly people in social, economic, cultural, and spiritual activities, helping them to maintain a large social network. Furthermore, the increased expenditure on pension, healthcare, and social care caused by an aging population may directly meet the great support demands of older people.^[59]^

### 4.4 Socioeconomic differences in older people's health and well-being

Tainan and Fuzhou are populated by the Chinese, but, as explained above, they differ in socioeconomic development and in social organization of work and community life. And the social support and SRH of older people in these 2 cities were significantly different. This can help to shed light on the impact of socioeconomic difference forces on older people’ health and well-being.

From the worldwide, the existence of wide socioeconomic differences in health and well-being shows how extraordinarily sensitive health and well-being remain to socioeconomic circumstances.^[60,61]^ At the domestic level, population ageing and changing health profiles are combining with rapid socioeconomic developments in mainland China and Taiwan would result in an increasing of social inequalities.^[62]^ The public health importance of socioeconomic inequalities in health and well-being at older ages is great because the socioeconomic inequalities may cause the relative risk of mortality translate into the absolute risk of mortality among people of lower SES.^[63,64]^ Evidence also indicates that socioeconomic inequalities in the absolute risk of mortality would obviously increase with age.^[65,66]^

First, sex and socioeconomic characteristics affect the incidence of disability or that risk factors are different between men and women among older people. Previous studies demonstrated that women were more likely to report poor SRH and to have a higher prevalence and incidence of disability compared with men at older ages.^[67–69]^ Other studies that were conducted in different countries also showed that women might have a higher risk of disability in their later years than men.^[70–72]^ Santosa et al have reported in low- and middle-income countries (LMICs) that women have longer life expectancy (LE) but proportionally less years of disability-free life expectancies (DFLEs) than men in different age groups among people older than 50 years, and are more evident in India, Ghana, Mexico, and the Russian Federation.^[73]^ Ng et al^[74]^ have confirmed the existence of sex differences in SRH in LMICs even after adjustments for differences in demographic and socioeconomic factors, and suggested that sex differences in health differed across the Health and Demographic Surveillance System (HDSS) sites, with the greatest level of inequality found in Bangladesh. The similar observations, putting women as the disadvantaged sex, have also been reported in some developed countries such as in the EU region, UK, and Japan.^[73]^ Emslie et al^[75]^ through drawing semistructured interview data from 45 men and women in 2 age cohorts (born in the early 1950 s and 1970 s) in the UK showed that multifactorial and sociocultural explanations were more common, more detailed, and less tentative than biological factors to explain the sex difference. However, Rodrigues et al^[76]^ found that after adjusted variations for sociodemographic, socioeconomic, and health factors, there was no difference between the sexes in the incidence of functional disability among elderly people, which may give us more clues to explore sex differences in health.

Second, socioeconomic gradients in health are associated with different material circumstances. Chaves et al^[77]^ found that higher income was associated with successful aging among healthy elders of southern urban area in Brazil. Demakakos et al^[78]^ showed wealth inequalities in mortality at older ages were sustained and wealth appeared to be more strongly associated with mortality than other socioeconomic position measures. Wilkinson^[79]^ illustrated that the mortality tented to be affected by relative material standards in developed countries and seemed to be lowest in countries that have small income difference. Alongside other socioeconomic factors, work itself may play an important part in generating social inequalities in health in working age. Marmot et al^[80]^ indicated that social differences in mortality based on an occupational status measure decrease after retirement, whereas those based on a non-work measure seem to decline less. As reported in this study, nonmanual worker scored the highest in social support, followed by blue-collar worker, peasants, and no stable works.

Third, some authors believed that education could be another good indicator of SES because it is relative fixed early adulthood. At older ages, income/wealth reflects the resources accumulated in the past that is highly related with individual health experiences.^[81]^ However, as people after 65 would tend to be out of the labor force, the effects of work on health may be indirected.^[82]^ Brown et al^[83]^ found that higher educational attainment has been associated with mortality compression in the United States. Bann et al^[84]^ reported that the effect of reducing incidence of mobility disability was larger for those with higher education or income among at-risk older adults aged 70 to 89 years.

Finally, it should be noted that the effects of social inequalities, particularly the higher levels of relative deprivation and lower social cohesion, may already be visible in mortality trends among young adults. Findings from the 2013/2014 Health Behaviour in School-aged Children (HBSC) survey reported that there is a positive picture of young people's health and behaviors, but the need to address existing social, age, and sex inequities persists.^[85]^ Many of the findings vary markedly across countries, reinforcing the importance of country-level factors and cultural norms to young people's health and well-being. Thus, social inequalities in young people’ health may be different compared with elderly people. Family relationships may be more changeable during the adolescent years especially for girls because the protective role of family may diminish while perceived support from friends remains relatively stable. Increasing use of mobile devices and media technology among young people can potentially open the door to increases in online/electronic aggression. School has an important influence on young people's lives. Younger children tend to have more positive experiences, although younger boys are more likely to experience school-related stress. However, adolescents from lower-affluence families tend to have poorer health, lower life satisfaction, higher levels of obesity and sedentary behaviors, poorer communication with their parents, less social interaction via social media, and lower levels of support from friends and family. Many of these inequalities are persistent and may be increasing.

### 4.5 Implications for social support improvement

We first recommend city government could participate in or learn from the WHO Healthy Cities project to protect and promote their citizens’ health and well-being.^[86]^ Inequality in health and urban poverty, vulnerable groups, and participatory governance as well as social determinants of health should be emphasized in the comprehensive health planning.

Second, health promotion may reduce the disparity in health status to help people to achieve their fullest health potential.^[87]^ Public health policy, supportive environment, community participation, personal ability, accessible health service, and moving into the future are the significant elements of health promotion action. In addition, different social, cultural, and economic systems should be taken into account while formulating any health promotion strategy and programme.

Third, developing social systems that provide support for the elderly through various economic, service, and mental health projects; this should be a focus of future work on aging. the US, health, wellness, mental health and other programs (particularly programs that are designed to support older low-income adults) have attempted to adapt and evolve in a changing social context that includes an increasing number of older people.^[88]^ In Norway, a series of successful work-related active aging programs were established, such as Prevention Programs, to increase wages and reduce working hours for older employees.^[89]^ In Thailand, the local government launched the Second National Plan for Older Persons (2002–2021), which focused on the development of policies and programs to support older people.^[90]^

Fourth, creating and improving a long-term care service system for the elderly may also increase their social support level, particularly disadvantaged older people. Tomita et al^[91]^ reported that in-home and community-based services would prevent hospitalization and institutionalization of individuals because it can improve the physical and mental state of individuals and reduce the care burden of caregivers. Ishibashi et al. found that home-help services could reduce the risk of functional decline among older people and help to facilitate their functional improvement.^[92]^ However, we should not ignore the socioeconomic inequalities when provide long-term to local elderly people.^[93]^

Fifth, encouraging participatory activities may improve support utilization levels.^[94,95]^ Pathways linking activities and individual well-being often include social support from social interactions, physical benefits from body movement, and the consequent positive psychological benefits.^[96,97]^ Many participatory programs for older people, such as village service in England, social activity formal support networks in the Philippines,^[98]^ leisure activities in Taiwan,^[99]^ and volunteer services in Europe and North America,^[100]^ have demonstrated that older people who participate in social activities have a corresponding increase in the level of their support utilization.

Finally, from a public health perspective, it is important to note that greater amounts of time engaged in PA,^[9]^ even at low intensity, would provide important benefits for maintaining physical function in old age.^[101,102]^ In addition, appropriate environment may increase outdoor mobility in older people. ^[103,104]^ Many studies have demonstrated that attractive and friendly environments, such as greens spaces, safe road crossings, and sidewalks, are closely related with higher PA among older individuals.^[105–108]^

### 4.6 Limitations

One limitation of the present study is its cross-sectional design, which precludes a causal sequence of social support and health. However, numerous current studies have demonstrated that causal relationship between social support and health is mixed; it is not easy to demonstrate this relation using a longitudinal design. Thus, our results may provide information concerning the similarities and differences in social support and health to better explore the relationship.

Another limitation is the participation source. Our participants were from single cities; therefore, it is not possible to generalize the conclusions for older people across all of Taiwan and Fujian Province. However, based on a comprehensive consideration of their economies, cultures, and social systems, Tainan and Fuzhou are relatively ideal cities in which to conduct this study. Therefore, further study designs can expand and include more comparable cities to reach deeper conclusions.

A third limitation is the questionnaire selection. Although the SSRS has been widely used among the elderly in Mainland China, this was the first time that it was used among Taiwan's older people. Lacking similar references in Taiwan, this scale may be too weak to describe the current social support of Taiwan's older people. However, this scale has been validated with high validity and reliability, and the purpose of the present study is not to test the feasibility of the SSRS in Taiwan but to compare the social support in the city of Tainan with that in Fuzhou in Mainland China. Accordingly, the scale does not have primary importance given that it is comparable across different studies in Mainland China.

In conclusion, the social support of Tainan's older people is high compared with the social support of Fuzhou's elderly. Age, education, marital status, living conditions, and SRH are the factors associated with social support among older people; therefore, potential interventions may need to be cohort-specific, with special attention paid to people of advanced age, with lower educational levels, single/divorced/widowed, living alone, and in poor health.

## Acknowledgements

The authors thank the professors at the Institute of Hospital and Health Care Administration of the Chia Nan University of Pharmacy and Science, the professors at the Institute of Environment Health of Fujian Medical University, and the professors in the Department of Social Medicine and Health Management and Department of Epidemiology and Statistics of Central South University for helping us complete this study.
